# Understanding factors associated with sarcopenic obesity in older African women from a low-income setting: a cross-sectional analysis

**DOI:** 10.1186/s12877-021-02132-x

**Published:** 2021-04-14

**Authors:** Amy E. Mendham, Julia H. Goedecke, Lisa K. Micklesfield, Naomi E. Brooks, Mieke Faber, Dirk L. Christensen, Iain J. Gallagher, Lillemor Lundin-Olsson, Kathryn H. Myburgh, Feyisayo A. Odunitan-Wayas, Estelle V. Lambert, Sebastiana Kalula, Angus M. Hunter

**Affiliations:** 1 SAMRC/Wits Developmental Pathways for Health Research Unit, Department of Paediatrics, School of Clinical Medicine, University of the Witwatersrand, Johannesburg, South Africa; 2 Research Centre for Health through Physical Activity, Lifestyle and Sport, Division of Exercise Science and Sports Medicine, Department of Human Biology, Faculty of Health Sciences, University of Cape Town, Cape Town, South Africa; 3 Non-communicable Diseases Research Unit, South African Medical Research Council Tygerberg, Cape Town, South Africa; 4 Health Sciences and Sport, University of Stirling, Stirling, Scotland; 5 Department of Public Health, University of Copenhagen, Copenhagen, Denmark; 6 Department of Community Medicine and Rehabilitation, Umeå University, Umeå, Sweden; 7 Department of Physiological Sciences, University of Stellenbosch, Stellenbosch, South Africa; 8 Division of Geriatric Medicine, University of Cape Town, Cape Town, South Africa

**Keywords:** Sarcopenia, Physical activity, Ageing, Diet, Inflammation, Food security

## Abstract

**Background:**

High rates of food insecurity, obesity and obesity-related comorbidities in ageing South African (SA) women may amplify the risk of developing sarcopenic obesity. This study aimed to investigate the prevalence and correlates of sarcopenic obesity and its diagnostic components [grip strength, appendicular skeletal muscle mass (ASM) and body mass index (BMI)] in older SA women from a low-income setting.

**Methods:**

This cross-sectional study recruited black SA women between the ages of 60–85 years (*n* = 122) from a low-income community. Testing included a fasting blood sample (markers of cardiometabolic risk, HIV), whole body and regional muscle and fat mass (dual-energy absorptiometry x-ray), anthropometry, blood pressure, functional movement tests, current medication use, demographic and health questionnaires, physical activity (PA; accelerometery), household food insecurity access scale, and a one-week quantified food frequency questionnaire. Foundation for the National Institutes of Health (FNIH) criteria (grip strength and ASM, adjusted for BMI) were used to classify sarcopenia. Participants with sarcopenia alongside a BMI of > 30.0 kg/m^2^ were classified as having sarcopenic obesity. Prevalence using other criteria (European Working Group on Sarcopenia in Older People, Asian Working Group for Sarcopenia and the International Working Group for Sarcopenia) were also explored.

**Results:**

The prevalence of sarcopenia was 27.9%, which comprised of sarcopenia without obesity (3.3%) and sarcopenic obesity (24.6%). Other classification criteria showed that sarcopenia ranged from 0.8–14.7%, including 0.8–9.8% without obesity and 0–4.9% with sarcopenic obesity. Using multivariate-discriminant analysis (OPLS-DA) those with sarcopenic obesity presented with a descriptive profile of higher C-reactive protein, waist circumference, food security and sedentary time than women without sarcopenic obesity (*p* = 0.046). A similar profile described women with low BMI-adjusted grip strength (*p* < 0.001).

**Conclusions:**

The majority of women with sarcopenia were also obese (88%). We show a large discrepancy in the diagnostic criteria and the potential for significantly underestimating the prevalence of sarcopenia if BMI is not adjusted for. The main variables common to women with sarcopenic obesity were higher food security, lower PA and chronic inflammation. Our data highlights the importance of addressing obesity within these low-income communities to ensure the prevention of sarcopenic obesity and that quality of life is maintained with ageing.

## Background

Economic and social transition in low-middle income countries (LMIC) has seen substantial increases in life expectancy alongside increasing rates of obesity and cardiometabolic disease [1]. Accordingly, age-related diseases such as sarcopenia are of increasing interest due to the impact on quality of life, frailty, falls and mortality [2–4]. Sarcopenia is operationally defined as low muscle mass and function, and has been shown to increase risk for developing functional limitations and physical disabilities, as defined by difficulty in performing daily activities [5]. Therefore, reversing, delaying, and/or preventing the onset of sarcopenia and maintaining functional mobility is paramount to ensuring quality of life with ageing [6]. However, data on the prevalence and understanding potential determinants of sarcopenia in LMIC's are scarce [7].

There are many different and population specific criteria for the classification of sarcopenia, including the European Working Group on Sarcopenia in Older People (EWGSOP [8, 9]), Asian Working Group for Sarcopenia (AWGS) [10], International Working Group for Sarcopenia (IWGS) [11] and The Foundation for the National Institutes of Health (FNIH) [7]. Development of the FNIH criteria included data in African Americans from a variety of income levels and has been previously used in studies of women from sub-Saharan Africa [7]. Importantly, the FNIH criteria incorporate cut-points for grip strength and appendicular skeletal muscle mass (ASM) adjusted for body mass index (BMI), which may be an optimal approach when classifying sarcopenia in individuals with obesity [12]. Notably, the criterion recommended for defining clinically meaningful low lean body mass is adjusted for BMI (ASM_BMI_) [12]. Moreover, individuals with obesity, regardless of age, have a greater absolute maximum muscle strength compared to individuals without obesity; however, when maximum muscular strength is normalised to body mass, individuals with obesity appear weaker [6]. Therefore, a similar approach by FNIH has identified clinically significant, gender-specific cut-points for BMI adjusted strength (Grip Strength_BMI_) [12, 13].

This is particularly relevant when assessing sarcopenia in LMIC's that present with an increasing double burden of malnutrition, which refers to the co-existence of undernutrition and overweight/obesity [14]. In particular, SA women have the highest prevalence of overweight and obesity in sub-Saharan Africa (68%) [15, 16], which is occurring simultaneously with 64% of households experiencing food insecurity [17]. Furthermore, food insecurity is an upstream determinant of behaviours such as diet and physical activity (PA), which are in turn associated with an increased risk of overweight and obesity [17, 18]. The presence of obesity coupled with sarcopenia has recently been termed ‘sarcopenic obesity’ or ‘sarcobesity’, and has been shown to exacerbate cardiometabolic risk and functional limitations [6, 19]. The determinants of sarcopenia and sarcopenic obesity is complex and incorporates multiple factors, such as physical inactivity, low protein intake, chronic systemic inflammation, insulin resistance and fat infiltration into the muscle [20–23]. Previous studies examining these determinants have occurred in cohorts from high-income countries [7–9, 11, 23–25], with little data available from low-income settings [7, 26, 27]. It is anticipated that high rates of food insecurity, obesity and obesity-related comorbidities in ageing SA women may amplify the risk of developing sarcopenic obesity. However, socioeconomic status, PA, diet, and cardiometabolic risk separating those with and without sarcopenia or sarcopenic obesity have not been previously explored in African women. Using a cross-sectional design, this study aimed to compare the prevalence of sarcopenia using different criteria and investigate the correlates of sarcopenic obesity and its diagnostic components (grip strength, ASM and BMI) in older SA women from a low-income setting.

## Material and methods

### Study participants

A convenience sample of community dwelling older women (*n* = 122) were recruited from a low-income, urban SA setting with a demographic profile composing of black South Africans [28]. Women were recruited from senior community groups/clubs and included those between the ages of 60–85 years, living independently (living in their own household or living with family) and who were ambulatory. One participant per household was recruited. Participants were excluded if they had any physical disability or condition that prevented them from completing the functional tests. In the context of the South African population, an older adult was classified as > 60 years based on the classification from the United Nations and due to the low life-expectancy of 65.1 years for South African adults (68.3 and 61.9 years for women and men, respectively) [29, 30].

### Study design and ethical consideration

For this cross-sectional observational study, clinical research workers visited the community centres for consent and screening after which participants attended the university-based laboratory on two separate occasions with one week between testing sessions. The first testing session included fasting blood sample and HIV testing, body composition, blood pressure, functional movement tests, and sociodemographic and health questionnaires. Participants were fitted with accelerometers and asked to complete sleep diaries for the following week, which were collected at the second testing session. Participants also completed a nutritionist-administered one-week food frequency questionnaire. This study was approved by the Human Research Ethics Committee of the Faculty of Health Sciences at the University of Cape Town (HREC REF:095/2018), and the NHS, Invasive or Clinical Research Committee at the University of Stirling (NICR:17/18). All participants provided written and verbal consent prior to testing procedures, including consent for HIV testing with counsellor support.

### Classification of sarcopenia and sarcopenic obesity

The FNIH Sarcopenia Project used an evidence-based approach to develop criteria for sarcopenia classification [12]. Recommendations for cut-points for low muscle strength and low lean muscle mass included Grip strength_BMI_ of < 0.56, and ASM_BMI_ of < 0.512, respectively. Participants presenting with both low Grip strength_BMI_ and ASM_BMI_ were classified as sarcopenic. Participants classified with sarcopenia alongside a BMI of > 30.0 kg/m^2^ were classified as having sarcopenic obesity [31]. The variability in the prevalence of sarcopenia and sarcopenic obesity based on the classification criteria of EWGSOP [8], EWGSOP-2 [9], AWGS [10], IWGS [11] and a criterion for ASMI in SA women [26] were also explored in this cohort (Table 1).

**Table 1:** Classification of participants according to different sarcopenia classification criteria

Variables	FNIH	EWGSOP	EWGSOP2	AWGS	IWGS	SA
Low ASMI (kg/m^2^)		27 (22.1)	27 (22.1)	17 (13.9)	16 (13.1)	3 (2.5)^a^
Low grip strength (kg)		63 (52.1)	24 (19.8)	25 (20.5)	–	–
Low gait speed (m/sec)		2 (1.6)	2 (1.6)	7 (5.7)	7 (5.7)	
Low ASM_BMI_	58 (47.5)					
Low grip strength_BMI_	56 (45.9)					
***Sarcopenia Classification***						
No sarcopenia	88 (72.1)	104 (85.3)	113 (92.6)	115 (94.3)	121 (99.2)	
Sarcopenia	34 (27.9)	18 (14.7)	9 (7.4)	7 (5.7)	1 (0.8)	
Sarcopenic non-obese	4 (3.3)	12 (9.8)	5 (4.1)	5 (4.1)	1 (0.8)	
Sarcopenic obesity	30 (24.6)	6 (4.9)	4 (3.3)	2 (1.6)	0 (0)	

Data reported as n(%). European Working Group on Sarcopenia in Older People, EWGSOP [8, 9]; The Foundation for the National Institutes of Health, FNIH [7]; Asian Working Group for Sarcopenia, AWGS [10]; International Working Group for Sarcopenia, IWGS [11]; South Africa, SA (Criteria for low muscle mass) [26]

^a^ No participants had low ASMI and obesity

## Components and correlates of sarcopenia and sarcopenic obesity

### Body composition

Body mass (BW-150, NAGATA, Tainan, Taiwan) and stature (3PHTROD-WM, Detecto, Missouri, USA) were measured in lightweight clothing for the calculation of BMI and used to classify obesity (> 30.0 kg/m^2^) [32]. Waist circumference (WC) at the umbilicus, and hip circumference (HC) at the largest protrusion of the buttocks, were measured using a metal anthropometric tape measure (*CESCORF*, Brazil). Circumferences were measured over the naked skin and noted to the nearest 0.1 cm. Circumferences were used to calculate waist to hip ratio (WHR), with abdominal obesity classified as WC > 80 cm and WHR > 0.85 [32].

Whole body composition was measured using dual energy x-ray absorptiometry (DXA; Discovery- W®, version 12.7.3.7, Hologic, Bedford, MA, USA) according to standard procedures. Sub-total (excluding the head) fat mass (FM) and fat-free soft tissue mass (FFSTM) were used for all analyses. Appendicular skeletal muscle mass (ASM) was calculated using the sum of FFSTM (kg) of both legs and arms, which was further adjusted for stature (ASMI; kg/height (m^2^)) and BMI (ASM_BMI_). DXA-derived regional body fat distribution, including android and gynoid were determined as previously described [33] and expressed as a percentage of subtotal FM. Peripheral (appendicular) fat (kg) was calculated as the sum of FM of both legs and arms, which was further divided by ASM to calculate peripheral fat/ASM ratio. Subcutaneous (SAT) and visceral (VAT) adipose tissue areas were estimated as described previously [34] and used to calculate VAT/SAT ratio. Bone mineral density was quantified using DXA at the spine (lumbar vertebrae L1-L5), total hip and femoral neck and World Health Organisation classification (t-score < − 2.5) was used to determine those with osteoporosis (Low t-score at > 1 site) [35].

### Sociodemographic and health questionnaires

Sociodemographic questionnaire included, estimated household monthly food costs, measures of household asset index (total of 11 assets that included the ownership of electrical appliances, computers, internet and/or motor vehicles), housing density (ratio of the number of people living in the house divided by the number of rooms) and the number of children (< 18 years) financially supported by the participants. Participants brought to the study site all medication/s currently prescribed to them for recording and was used to identify disease prevalence of hypertension, dyslipidemia, diabetes, arthritis, and HIV. Disease prevalence for cancer and tuberculosis was obtained using self-reported diagnosis and medication history of the disease and osteoporosis was identified from bone mineral density scans (DXA). Notably, through verbal communication with participants we identified a low reliability in the self-reported patient history of several chronic conditions (i.e. stroke, heart failure and coronary artery disease), which are not reported in the current study. Multi-morbidity was classified as having 2 or more of the aforementioned listed diseases. All participants underwent a rapid HIV test, due to previously identified associations between HIV and premature ageing, sarcopenia, obesity and visceral adiposity [36, 37]. Pre and post-test counselling were completed by a trained HIV counsellor and if the participant was HIV positive (known or unknown) they continued in the study and were referred to a local clinic for follow-up testing and counselling. All information on current and past smoking (duration of smoking and number of daily cigarettes) behaviours was obtained. Participants were also asked if they had fallen in the past year. A fall was defined as ‘any time you unexpectedly (or unintentionally) landed on the floor or ground’ [38]. Participants further completed a Household Food Insecurity Access Scale (HFIAS), which consists of nine questions about worry, availability and accessibility of foods for the household during the previous 30 days [39]. Total HFIAS ranges from 0 (food security) to 27 (maximum food insecurity).

### Components of metabolic syndrome, inflammation, iron and vitamin-D

Participants arrived at the laboratory at 09:00 following an overnight fast (10–12 h). A fasting venous blood sample was collected for the analysis of cardiometabolic risk markers. Specifically, components of metabolic syndrome, including glucose (enzymatic method with hexokinase, Cobas Analyzer, Roche Diagnostics, Basel, Switzerland), high-density lipoprotein (HDL) cholesterol, and triglyceride (enzymatic colorimetric test, Cobas Analyzer) concentrations were analysed. Metabolic syndrome was determined if participants had three or more components using the 2009 harmonized criteria [40]. These components included: elevated waist circumference (≥80 cm in women), elevated triglycerides (≥1.7 mmol/L and/or using cholesterol lowering medication), reduced HDL cholesterol (< 1.3 mmol/L in women), elevated blood pressure (≥130 mmHg for systolic and/or ≥ 85 mmHg for diastolic and/or using blood pressure medication), and elevated glucose (≥5.6 mmol/L and/or using diabetes medication). Vitamin- D 25 OH (electrochemiluminescence binding assay, Cobas Analyzer), C-Reactive protein (CRP) (high-sensitivity, particle enhanced immunoturbidimetric assay, Cobas Analyzer) and ferritin (immunoassay, Cobas) were analysed. An acute inflammatory response was suspected when a high ferritin level (> 150 ng/mL) coincided with high CRP (> 20 mg/dL) and these data (*n* = 11) were removed from analyses [41].

Blood pressure was measured 3 times at 1-min intervals using an appropriately sized cuff and an automated blood pressure monitor (Omron 711, Omron Healthcare, Hamburg, Germany), after participants had rested in a seated position for 30 min. The mean of the two measurements is presented.

### Functional movement tests

All functional movement tests were conducted on the same day and by the same research assistant. All tests were conducted in the order reported below.

#### Strength

Grip strength (kg) was measured in a seated position on the non-dominant hand using a hand dynamometer (T.K.K. 5401, Grip-D, Takei, Tokyo, Japan). The measures were taken with the arm static and elbow by the side in a right-angle position. The test was repeated 3 times with a 1-min rest between tests. The maximum score was used in the analysis [42].

#### Gait speed

Participants were required to complete a 10-m walk test which involved walking ‘at a fast pace’ between two markers that were set up 14 m apart. Tape was placed on the ground at the 2-m and 12-m mark in order to exclude the acceleration and deceleration phase to ensure a measure of ‘steady state’ walking was obtained [43]. The time taken to walk between the 2- and 12-m markers was recorded, and the test was repeated twice with a 1-min rest between tests. The mean score was used in the analysis (m/s).

#### Agility/dynamic balance

The 3-m timed-up and go test was completed by measuring the time taken to get up from a seated position (without using arms for assistance), walk around a marker at 3 m, and return to the seated position. This test was repeated twice with a 1-min rest between tests. The fastest time was used in the analysis (seconds) [44].

#### Aerobic endurance

The 6-min walk test was conducted once to measure the number of metres covered by walking in a 6-min time period. The research assistant used a stopwatch and walked quietly behind the participant during the test to mark and monitor the distance covered. Participants walked in a 20 × 5 m rectangle [43].

#### Physical activity, sleep and sedentary behaviour

Physical activity, sleep and sedentary behaviour were measured simultaneously with an Actigraph (GTX3+, ActiGraph LLC, Pensacola, Florida) and ActivPAL (PAL Technologies Ltd., Glasgow, Scotland). Both accelerometers were worn for seven consecutive 24-h days. The Actigraph was attached to the waist with a lightweight belt and the ActivPAL was worn on the mid anterior right thigh. Participants completed a sleep diary to record wake and sleep times over the 7-day period. Actigraph data was analysed (ActiLife, version 6) with valid wear defined as 600 min of waking wear time per day for a minimum of four days. Troiano cut-points were used to define total PA (> 100 cpm), light PA (100–2019 cpm) and moderate to vigorous physical activity (MVPA; > 2020 cpm) [45]. ActivPAL data were analysed (CREA beta-algorithm, PAL analysis, Version 8.10.8.32) with all participants presenting with a minimum of seven consecutive 24-h days. Wear time included a 24-h protocol, allowing for 4 h of non-wear time, minimum of 10 s non-upright and upright periods. Daily step count and number of sit to stand transitions, time spent upright (total of standing and stepping time), standing, stepping sitting, napping (secondary lying time), sleeping (primary lying time), total time spent in sedentary bouts of more than 30 and 60 min are reported, and daily energy expenditure (calories) was calculated by multiplying daily metabolic equivalent of task (MET) by body weight (kg).

#### Dietary intake

A nutritionist administered a 7-day quantified food frequency questionnaire (QFFQ) for each participant. Food portion sizes were estimated using household utensils, food containers and packaging, three-dimensional sponge models and “dish-up and measure”. Amounts reported in household measures or volume were converted to grams using the SA Medical Research Council (SAMRC) Food Quantities Manual for SA [46]. Food intake was converted to energy and macronutrients (fats, carbohydrates, protein (animal and plant), fibre, and added sugar) using the SA food composition database [47]. Food consumed during the 7-day reference period for the QFFQ were categorized into 12 food groups and expressed as a % of energy intake (%EI), based on a recent SA study [48]. The food groups included, fruits, vegetables, cooked porridge, starchy grains, legumes, nuts and seeds, milk and dairy products, animal source foods, fats and oils, sugar and sugary foods, savoury snacks, dishes and sauces and alcohol. Dietary data was removed if the participant reported below 4000 kJ per day (*n* = 19 removed).

### Sample size calculation and statistical analyses

Based on methodology in Kruger et al. [26] we used G*Power 3.1 to estimate sample size for a multiple regression model with a medium effect size (F tests – Linear multiple regression: Fixed model, R^2^ increase at 95% power, medium effect size = 0.15), which gave a sample size of *n* = 107.

Data were expressed as mean ± standard deviation (SD) or median interquartile range (IQR - 25th–75th percentile) depending on the normality of continuous variables. Normality was tested using Shapiro-Wilks test and skewed data were log transformed before analysis. Data was analysed using IBM SPSS statistics (Version 26, Statistical Package for the Social sciences, Chicago, IL, USA). *P* values are uncorrected for multiple testing and are reported, with significance set at *p* < 0.05.

All outcomes were firstly compared univariately between groups (with and without sarcopenia) using an independent t-test or Mann-Whitney U test for normally distributed and skewed data, respectively. Secondly, to investigate the differences in lifestyle behaviors (diet and PA), components of metabolic syndrome, body composition, functional movement and socioeconomic status between those with and without sarcopenic obesity, we explored the data using a multivariate analysis. Firstly, a principle component analysis identified that there were no outliers. Secondly, orthogonal partial least squares discriminant analysis (OPLS-DA; SIMCA v.15.2 (Sartorius, Umetrics, Umea, Sweden) models were calculated to explore the differences between those with and without sarcopenic obesity. OPLS-DA is a supervised modelling approach that uses a predefined binary variable as the outcome (2 class model that describes those with and without sarcopenic obesity). Finally, two further OPLS models were conducted using grip strength_BMI_ and ASM_BMI_ as continuous predictors. Both OPLS and OPLS-DA models summarise the largest systematic variation in the dataset into 1 latent variable (OPLS as one constant predictor variable and OPLS-DA as a 2-class model). These methods are highly suitable for a large number of highly correlated variables and provides information on the variables that have the largest discriminatory power [49]. To prevent overfitting of models, the models were validated based on ANOVA of the cross-validated OPLS-DA scores (CV-ANOVA) for significance testing [50]. A validated and significant model was considered with a CV-ANOVA of *p* < 0.05. Variables in each model were considered significant when fulfilling the statistical significance criteria using post-hoc linear regression on loadings calculated from the validated OPLS-models on a 95% confidence level [51]. All data in the OPLS models are reported as loading weight (w) with 95% confidence intervals, which describes the contribution of each listed variable (X variable) to the model. Variables with large w’s (positive or negative) are highly correlated with the Y variable that represents the continuous predictors (OPLS) or groupings (OPLS-DA).

## Results

### Sarcopenia criteria

The application of different sarcopenia criteria is presented in Table 1. The FNIH criteria for sarcopenia was used to classify participants with sarcopenia (27.9%), which comprised of sarcopenia without obesity (3.3%) and sarcopenic obesity (24.6%). Using other sarcopenia classification criteria, the prevalence of sarcopenia ranged from 0.8–14.7%, which included 0.8–9.8% without obesity and 0–4.9% with sarcopenic obesity.

We then compared functional and body composition characteristics of the women with and without sarcopenia based on the FNIH criteria (Table 2). The majority (*n* = 87, 71%) of the total sample were classified as obese, with 88% (*n* = 30) of participants with sarcopenia and 65% (*n* = 57) of participants without sarcopenia being obese (*p* = 0.005). Accordingly, variables relating to total adiposity and body fat distribution were higher in those with sarcopenia (*p* < 0.05). Functional movement tests showed lower grip strength, gait speed and endurance (6-min walk test) in those with sarcopenia (*p* < 0.05).

**Table 2:** Body composition, fat distribution and functional movement characteristics of the cohort and those with and without sarcopenia

Variables	Cohort (***n*** = 122)	No sarcopenia (***n*** = 88)	Sarcopenia (***n*** = 34)	***P*** value
Age (Years)	67 (64–71)	67 (64–71)	68 (64–71)	0.943
Height (cm)	155.8 ± 6.1	157.0 ± 6.1	152.9 ± 4.9	0.001
Weight (kg)	81.6 (69.0–98.9)	77.1 (66.6–91.8)	92.4 (81.7–108.9)	0.002
BMI (kg/m^2^)	33.1 (29.0–40.1)	31.9 (28.3–35.6)	39.3 (34.3–45.4)	< 0.001
Waist-to-hip ratio (cm)	0.95 (0.87–0.10)	0.95 (0.88–0.99)	0.96 (0.84–1.0)	0.945
Body fat-mass (kg)	36.6 (28.2–47.6)	32.7 (26.0–41.9)	47.4 (37.7–55.9)	< 0.001
Body fat-mass (%)	48.6 ± 5.9	47.0 (43.8–50.6)	55.0 (51.0–57.0)	< 0.001
FFSTM (kg)	36.7 (32.6–41.3)	35.7 (32.8–41.1)	37.5 (32.3–42.9)	0.733
Android (%)	8.6 ± 1.4	8.6 ± 1.5	8.7 ± 1.5	0.880
Gynoid (%)	16.0 ± 2.5	16.1 ± 2.5	15.6 ± 2.2	0.260
VAT (cm^2^)	196.3 ± 74.5	183.4 ± 75.0	229.0 ± 65.0	0.002
SAT (cm^2^)	477.9 ± 138.2	441.2 ± 132.9	569.7 ± 105.6	< 0.001
VAT/SAT ratio	0.40 (0.32–0.48)	0.42 ± 0.13	0.40 ± 0.10	0.628
ASM (kg)	16.5 (14.7–19.6)	16.5 (14.6–19.5)	17.4 (15.0–20.2)	0.455
ASMI (kg/m^2^)	6.9 (6.1–7.9)	6.8 (6.0–7.5)	7.4 (6.2–8.8)	0.036
ASM_BMI_ (kg/m^2^)	0.514 (0.459–0.578)	0.54 (0.50–0.67)	0.45 (0.42–0.48)	< 0.001
Peripheral fat (kg)	18.2 (14.0–23.9)	16.3 (13.4–20.4)	24.7 (19.5–28.2)	< 0.001
Peripheral fat/ASM ratio	1.1 ± 0.3	1.01 ± 0.20	1.36 ± 0.20	< 0.001
***Functional Movement***				
Grip strength (kg)	19.6 ± 4.5	20.4 ± 4.3	17.6 ± 4.3	0.002
Grip strength_BMI_ (kg/m^2^)	0.59 ± 0.18	0.63 (0.55–0.76)	0.48 (0.39–0.53)	< 0.001
6-min walk (m)	450 (395–490)	462.5 (402.5–496.3)	405.0 (351.4–462.3)	0.010
Gait speed (m/sec)	1.53 (1.38–1.67)	1.55 (1.41–1.68)	1.46 (1.31–1.56)	0.022
3 m timed-up and go (sec)	6.9 (6.2–8.1)	6.7 (6.1–7.8)	7.2 (6.5–8.4)	0.057

All normally distributed and skewed data are reported as mean ± SD and Median (IQR – 25-75th percentile). *Abbreviations*: *BMI* Body mass index, *FFSTM* Fat-free soft tissue mass, *VAT* visceral adipose tissue, *SAT* subcutaneous adipose tissue, *ASM* appendicular skeletal muscle mass, *ASMI* appendicular skeletal muscle mass index, *ASM*_*BMI*_ appendicular skeletal muscle mass adjust for body mass index, *Grip* *strength*_*BMI*_ Grip strength adjusted for body mass index

*P* values represent a significant difference between those with and without sarcopenia. Parametric and non-parametric (Mann-Whitney U) independent t-tests were conducted on normally distributed and skewed data, respectively

Univariate analysis shows that the majority of the cohort (73%) presented with metabolic syndrome and the prevalence tended to be higher in women with sarcopenia (Table 3). Furthermore, the sarcopenic group had higher CRP and lower iron levels (ferritin) compared to those without sarcopenia (*p* < 0.05). Participants with sarcopenia supported more children (*p* < 0.05) and few women in the cohort were previous (*n* = 5) or current smokers (*n* = 11).

Physical activity, sedentary behaviour and dietary intake data are shown in Table 3. Univariate analysis showed that women with sarcopenia presented with a lower daily step count, stepping time, and sit to stand transitions than women without sarcopenia (*p* < 0.05). The mean macronutrient consumption for the whole cohort comprised of 64.8 ± 7.2%EI carbohydrates, 12.3 ± 2.1%EI protein and 21.2 (17.5–25.1) %EI fat. Further, the dominant food groups consumed were starchy grains (31.1 ± 11.4%EI), and sugar and sugary foods (14.9 (10.1–20.8) %EI). There were no differences in dietary intake between the women with sarcopenia and those without.

**Table 3:** Lifestyle behaviours, components of metabolic syndrome and sociodemographic characteristics of the cohort and those with and without sarcopenia

Variables	Cohort	No sarcopenia	Sarcopenia	***P*** value
***Components of metabolic syndrome, inflammation, iron and vitamin D***	***N*** **= 121**	***N*** **= 88**	***N*** **= 33**	
Metabolic Syndrome n(%)	89 (73.0)	60 (68.2)	29 (85.3)	0.047
Waist Circumference (cm)	99.7 ± 15.1	97.1 ± 14.8	106.4 ± 13.7	0.002
Diastolic Blood Pressure (mmHg)	71.2 (64.7–78.9)	70.5 ± 12.1	74.3 ± 11.2	0.059
Systolic Blood Pressure (mmHg)	131.5 (118.9–172.7)	131.5 (119.1–148.4)	130.7 (117.7–146.0)	0.873
Glucose (mmol/L)	5.1 (4.8–6.4)	5.0 (4.7–6.4)	5.2 (4.8–6.2)	0.517
Triglycerides (mmol/L)	1.1 (0.8–1.6)	1.1 (0.8–1.6)	1.2 (1.0–1.8)	0.049
High-Density Lipoprotein (mmol/L)	1.3 (1.1–1.6)	1.3 (1.1–1.6)	1.3 (1.1–1.5)	0.440
C-Reactive Protein (mg/L)*	5.7 (2.2–8.7)	4.1 (1.9–7.4)	7.9 (4.2–12.8)	< 0.001
Ferritin (ng/mL)*	112.1 (63.2–180.4)	127.2 (73.1–196.3)	85.0 (54.1–135.9)	0.014
Vitamin D-25OH (ng/mL)	13.0 (11.0–17.0)	13.0 (11.0–17.0)	13.0 (10.8–16.8)	0.771
***Sociodemographics***	**N = 122**	**N = 88**	**N = 34**	
Asset Index (n)	8 (6–9)	8 (6–9)	8 (6–9)	0.599
Children supported in household (n)	1 (0–3)	1 (0–2)	2 (1–3)	0.024
Housing Density	1.0 (0.6–1.4)	1.0 (0.6–1.3)	1.0 (0.6–1.8)	0.474
Food Insecurity (HFIAS)	6 (3–11)	7 (3–13)	6 (3–8)	0.088
Estimated Monthly Food Cost (US$)	90 (67–167)	90 (60–127)	93 (67–122)	0.738
Previous Smoker n(%)	5 (4.1)	2 (2.3)	3 (8.8)	0.036
Current Smoker n(%)	11 (9.0)	11 (12.5)	0 (0.0)	0.145
***Physical activity and sedentary behaviour***	***N*** **= 116**	***N*** **= 83**	***N*** **= 33**	
Daily light PA (%)	37.1 ± 9.3	37.9 ± 9.8	35.0 ± 7.4	0.127
Daily MVPA (%)	1.0 (0.3–1.8)	1.0 (0.3–1.8)	0.95 (0.2–1.9)	0.765
Light PA (min/day)	326.2 ± 91.0	335.1 ± 94.9	303.4 ± 76.9	0.097
MVPA (min/day)	9.1 (2.3–15.9)	9.1 (2.5–15.9)	8.4 (1.4–15.8)	0.757
Daily Step count (n)	6848 (5122–8784)	7574 ± 3417	6037 ± 2431	0.020
Upright (min/day)	415.3 ± 125.9	422.8 ± 126.5	396.5 ± 124.5	0.312
Standing (min/day)	317.4 ± 107.0	319.6 ± 104.7	311.7 ± 114.2	0.720
Stepping (min/day)	97.9 ± 38.7	103.1 ± 40.9	84.8 ± 29.0	0.021
Sitting (min/day)	402.4 ± 113.4	401.1 ± 106.6	405.7 ± 130.7	0.973
Napping (min/day)	28.3 (0.0–64.1)	35.4 (0–67.4)	28.2 (13.7–51.8)	0.674
Sleeping (min/day)	568.6 (499.7–629.1)	566.1 (499.5–629.8)	582.3 (499.4–629.8)	0.592
Sitting bouts > 30 min (n/day)	4 ± 1	4 ± 1	4 ± 2	0.414
Sitting bouts > 60 min (n/day)	1 (1–2)	1 (1–1)	1 (1–2)	0.206
Sitting time > 30 min (min/day)	213.8 ± 92.3	206.5 ± 84.6	232.3 ± 108.6	0.175
Sitting time > 60 min (min/day)	92.8 (51.1–161.8)	89.5 (51.0–147.8)	104.7 (52.8–192.1)	0.259
Sit to stand transitions (n/day)	38 ± 12	40 (32–46)	33 (26–42)	0.007
***Dietary intake: Macronutrients***	***N*** **= 103**	***N*** **= 74**	***N*** **= 29**	
Energy Intake (KJ)	7646 (5994–9991)	7870 (6057–10,361)	7462 (5903–8678)	0.278
Carbohydrates (%EI)	64.8 ± 7.2	64.8 ± 7.0	64.9 ± 7.7	0.940
Protein (%EI)	12.3 ± 2.1	12.3 ± 2.0	12.5 ± 2.5	0.648
Animal Protein (%EI)	5.8 ± 2.4	5.7 (4.0–7.7)	5.4 (4.1–7.7)	0.883
Plant Protein (%EI)	6.5 ± 1.5	6.4 (4.5–7.3)	6.3 (5.3–7.6)	0.797
Total Fat (%EI)	21.2 (17.5–25.1)	21.3 (17.4–25.1)	21.2 (17.1–24.3)	0.843
PUFA (%EI)	5.7 (4.1–7.5)	5.7 (4.1–7.4)	5.7 (4.0–8.1)	0.889
MUFA (%EI)	6.7 (5.2–8.1)	6.7 (5.1–8.1)	6.6 (5.3–8.2)	0.982
Saturated Fat (%EI)	6.5 ± 2.2	6.5 ± 2.3	6.6 ± 2.2	0.901
Added sugar (%EI)	12.7 (8.6–18.0)	11.7 (8.4–18.1)	13.3 (9.0–18.0)	0.585
Cholesterol (g/4200 KJ)	73.7 (53.2–109.8)	72.7 (52.6–108.9)	88.4 ± 46.9	0.730
Fibre (g/4200 KJ)	14.1 (11.6–17.0)	14.1 (11.6–16.8)	13.9 (11.6–17.3)	0.708
***Dietary intake: Food Groups***	***N*** **= 110**	***N*** **= 81**	***N*** **= 29**	
Milk and Dairy products (%EI)	7.2 (3.6–12.4)	7.8 (3.5–12.4)	7.0 (3.6–12.6)	0.585
Fats and oils (%EI)	3.4 (1.2–6.4)	3.5 (1.3–6.2)	3.0 (1.0–8.0)	0.916
Animal protein foods (%EI)	9.4 (5.7–13.6)	9.0 (5.5–13.1)	10.1 (6.1–14.9)	0.362
Fruit (%EI)	6.4 (3.0–11.1)	6.1 (3.0–10.8)	7.2 (2.9–13.6)	0.900
Vegetables (%EI)	2.6 (1.6–4.9)	2.6 (1.5–4.8)	2.4 (1.7–5.9)	0.585
Legumes (%EI)	1.7 (0.0–3.6)	1.8 (0.0–3.1)	1.5 (0.0–5.2)	0.758
Cooked Porridge (%EI)	8.4 (5.0–15.8)	9.0 (4.3–16.5)	7.8 (5.1–14.9)	0.732
Starchy grains (%EI)	31.1 ± 11.4	31.4 ± 10.7	30.1 ± 13.4	0.596
Savoury snacks, dishes and sauces (%EI)	0.8 (0.0–2.0)	0.8 (0.0–2.3)	0.7 (0.1–1.8)	0.948
Sugar and sugary foods (%EI)	14.9 (10.1–20.8)	14.8 (9.6–21.2)	15.1 (10.4–19.8)	0.922
Nuts and seeds (%EI)	0 (0–1.9)	0 (0–2.0)	0 (0–1.8)	0.905
Alcohol (%EI)	0 (0–0)	0 (0–0)	0 (0–0)	0.225

All normally distributed are reported as mean ± SD and and skewed data as median (IQR – 25-75th percentile). *Abbreviation*: *HFIAS* Household food insecurity access scale, *PA* Physical activity, *EI* Energy intake, *MVPA* Moderate-to-vigorous physical activity, *MUFA* Monounsaturated fatty-acids. *P* values represent a significant difference between those with and without sarcopenia. Parametric and non-parametric (Mann-Whitney U) independent t-tests were conducted on normally distributed and skewed data, respectively. *CRP, cohort (*n* = 104), no sarcopenia (*n* = 73), sarcopenia (*n* = 33); Ferritin, cohort (*n* = 111), no sarcopenia (*n* = 81), sarcopenia (*n* = 33)

The prevalence of falls and multimorbidity are presented in Table 4. Falls in the last year were reported by 38% (*n* = 46) of women and this did not differ between those with and without sarcopenia (*p* > 0.05). Ninety one percent (*n* = 111) of women had multimorbidity (> 2 diseases) and this did not statistically differ by group (p > 0.05). Women with sarcopenia had a higher prevalence of hypertension (97%, *n* = 33) compared to women without sarcopenia (82%, *n* = 72; *p* < 0.05).

**Table 4:** History of falls and prevalence of chronic diseases and multimorbidity

Variables	Cohort (***n*** = 122)	No sarcopenia (***n*** = 88)	Sarcopenia (***n*** = 34)	***P*** value
Fallen in past year	46 (38.0)	34 (38.6)	12 (36.4)	0.819
Hypertension	105 (86.1)	72 (81.8)	33 (97.1)	0.029
Dyslipidemia	89 (73.0)	63 (71.6)	26 (81.3)	0.586
Diabetes	44 (36.1)	32 (32.7)	12 (35.3)	0.912
Arthritis	26 (21.3)	18 (20.5)	8 (23.5)	0.710
Osteoporosis	29 (23.8)	22 (25.0)	7 (20.6)	0.608
History of cancer	3 (2.5)	1 (1.1)	2 (5.9)	0.129
HIV	9 (7.4)	7 (8.0)	2 (5.9)	0.695
History of tuberculosis	22 (18.0)	19 (21.6)	3 (8.8)	0.100
Multi-morbidities (> 2 diseases)	111 (91.0)	81 (92.0)	30 (88.2)	0.501

All data reported as n (%). Chi-square was used to determine differences in frequency of each variable between those with and without sarcopenia. *Abbreviation*: *HIV* human immunodeficiency virus

### Correlates of sarcopenic obesity and its components using OPLS modelling

**Fig. 1:**
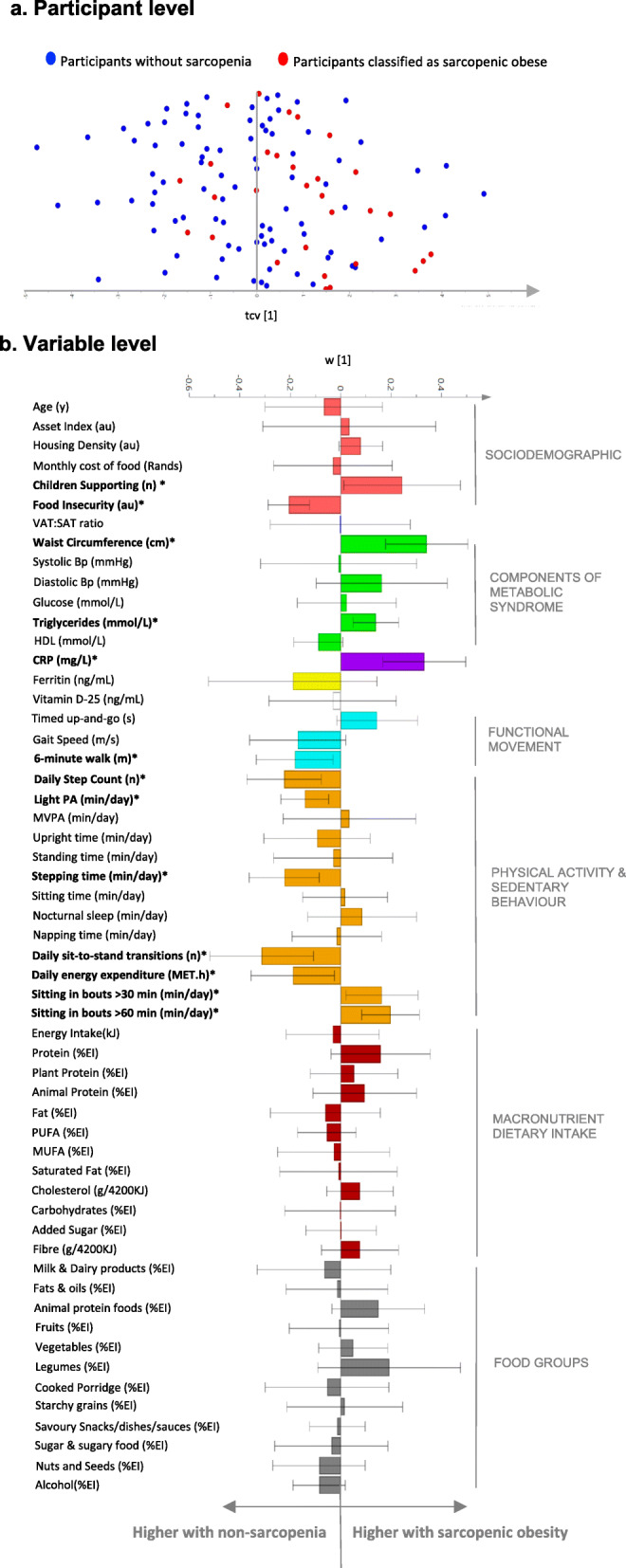
*Multivariate characteristic profile for non-sarcopenic and sarcopenic obese participants (n = 118; OPLS-DA CV-ANOVA P < 0.001).* **(a)** *OPLS-DA cross-validated scores (tcv [1]) that describes participant variability in the characteristic profile.* **(b)** The characteristic profile that discriminates between non-sarcopenic and sarcopenic obese participants. The asterisk (*) represent variables that significantly (P < 0.05) discriminate between those with and without sarcopenic obesity. Error bars represent 95% confidence intervals. All data in the OPLS-DA models are reported as loading weight (w [1]), which describes the contribution of each listed variable (X variable) to the model

As the majority of participants with sarcopenia (FNIH criteria) were also obese we explored correlates of sarcopenic obesity. OPLS-DA modelling was used to determine a profile of variables that discriminated between non-sarcopenic and sarcopenic obese individuals (CV-ANOVA *p* < 0.001; Fig. 1). Figure 1 (a) presents individual variability based on group classification (b) and the variables that significantly discriminated between groups. This model presents behavioural (dietary intake, PA and sedentary behaviour), sociodemographic and phenotypic characteristics (components of metabolic syndrome, VAT/SAT ratio and functional movement) to assist in developing a profile in SA women that may identify those with sarcopenic obesity. Interestingly, those with sarcopenic obesity supported more children and were more food secure. Unsurprisingly, several metabolic syndrome components (waist circumference and triglycerides), alongside chronic systemic inflammation (CRP) were higher in those with sarcopenic obesity. In terms of the functional movement tests, gait speed was not a significant contributor to the model, but rather those with sarcopenic obesity had lower cardiovascular fitness (6-min walk test), daily step count, stepping time, light PA, sit to stand transitions, and daily energy expenditure. Time spent sitting for more than 30 and 60-min bouts was significantly higher in those with sarcopenic obesity. Interestingly, there were no dietary intake characteristics that significantly contributed to the sarcopenic obesity profile.

**Fig. 2:**
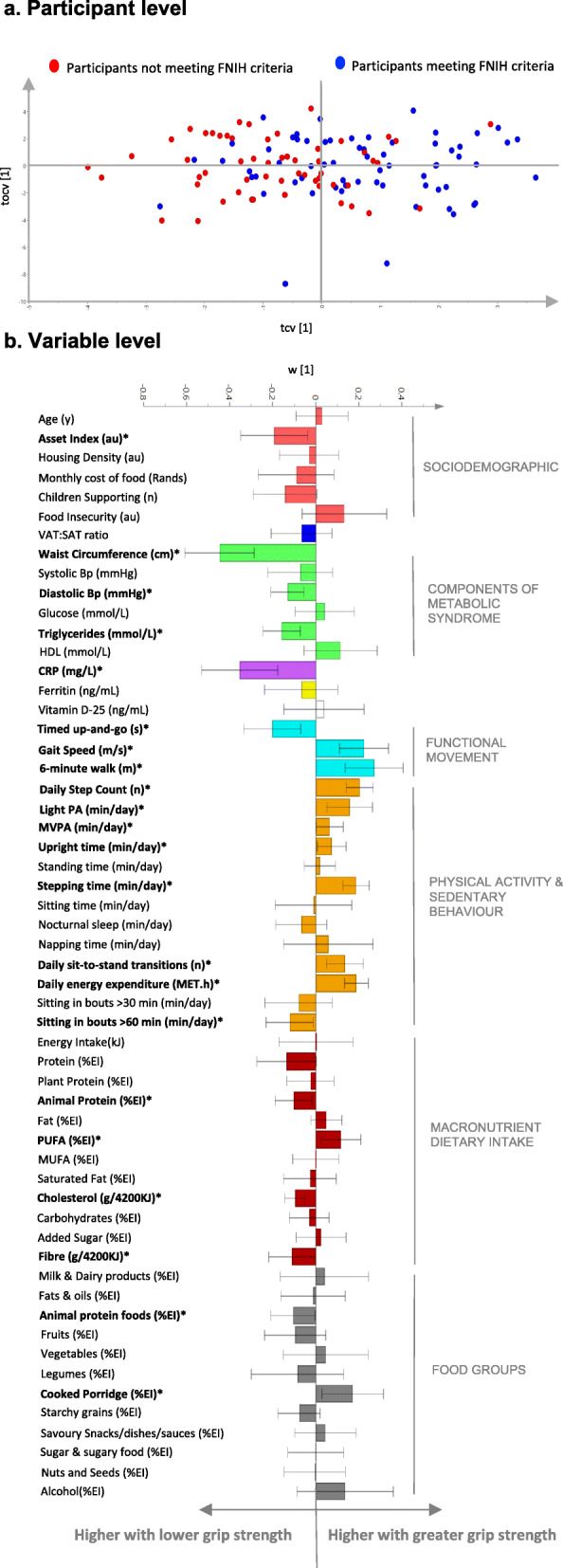
*Multivariate associations between changes in BMI-adjusted grip strength (FNIH criteria) and characteristics that relate to the pathophysiology of sarcopenia (n = 121; OPLS CV-ANOVA P < 0.001).* **(a)** *OPLS cross-validated scores (tcv [1]) that describes participant variability and* ***(b)*** multivariate associations in the characteristic profile at the variable level. The asterisk (*) represent variables that are significantly (P < 0.05) associated grip strength. Error bars represent 95% confidence intervals. All data in the OPLS models are reported as loading weight (w [1]), which describes the contribution of each listed variable (X variable) to the latent variable that is produced by the model. Variables with large weights (w [1]) (positive or negative) are highly correlated with grip strength

The two main components for the FNIH classification of sarcopenia used in the current study include, ASM_BMI_ and grip strength_BMI._ To understand the contribution of each correlate to the latent variable we generated two OPLS models with ASM_BMI_ and grip strength_BMI_ as continuous predicted variables. Notably, there was no significant OPLS model profile that correlated with ASM_BMI_ (CV-ANOVA *p* = 1.00). In contrast, Fig. 2 (a) shows individual variability in the model and identifies participants with low grip strength_BMI_ based on the FNIH criteria. The significant cross-validated model (CV-ANOVA *p* < 0.001; Fig. 2 b) identifies that a higher asset index was the only significant sociodemographic component that was associated with lower grip strength_BMI._ In terms of the components of metabolic syndrome, a lower grip strength_BMI_ was associated with a higher waist circumference, blood pressure, triglycerides and systemic inflammation (CRP). Those with a lower grip strength_BMI_ had lower gait speed, agility and balance (TUG) and cardiovascular fitness (6-min walk test). This model shows a similar PA profile that identified those with sarcopenic obesity, with lower daily MVPA and time spent upright as additional variables that were associated with a lower grip strength_BMI_. In terms of dietary intake, consumption of cooked porridge (%EI) correlated with a higher grip strength_BMI_, while consumption of animal protein foods (%EI), cholesterol and fibre correlated with a lower grip strength_BMI._

## Discussion

In this sample of older African women from a low-income setting, we show a 27.9% prevalence of sarcopenia, which comprises of 3.3% with sarcopenia and 24.6% with sarcopenic obesity. This highlights that both sarcopenia and obesity co-exist in this cohort, with the FNIH criterion reporting a significantly higher prevalence than when using other age and population specific cut-points. These findings indicate a large discrepancy in diagnostic criteria and the potential for significantly underestimating the prevalence of sarcopenia if an appropriate population-specific criterion is not applied and if BMI is not adjusted for. Furthermore, we show that the main correlates that describe a profile of sarcopenic obesity were lower food insecurity, low PA and fitness, and a chronic inflammatory state; highlighting the importance of addressing obesity within these communities to prevent sarcopenic obesity and maintaining quality of life with ageing.

The majority of women classified as sarcopenic (27.9%) were also obese (88%). However, the current study also shows that other criteria (EWGSOP, IWGS and AWGS) present a prevalence of total sarcopenia which ranged from 0.8–14.7%, which indicates the need for a population-specific criterion that has not been previously addressed in Africa. Importantly, the FNIH criteria include data in African American cohorts and were generated from data providing normative values for grip strength and ASM when adjusting for BMI [12]. Previous findings have shown that sarcopenia defined using methods considering both stature and fat mass are better at predicting weakness, reduced physical function and overall sarcopenia related disability than using ASMI and grip strength methods alone [3, 6, 12, 13]. Given the high prevalence of obesity in the current cohort (71%) and SA women (overweight and obesity of ~ 68%) [15, 16], this factor should not be ignored when assessing sarcopenia in this setting. Indeed, these data clearly indicate that FNIH BMI adjusted criteria may be an optimal set of criteria [15, 16, 31]. Previous data using the EWGSOP in SA women has shown a prevalence of 8.9% sarcopenia, however, this study included younger (45+ years old) women with a BMI range of underweight to overweight [27]. Using the same criteria (Table 1) our cohort reports a prevalence of 14.7%. Furthermore, a Gambian cohort of women between 40 and 75+ years, with normal BMI (~ 22 kg/m^2^) reported a prevalence of 45% and 10% sarcopenia when applying FNIH and EWGSOP criteria, respectively [7]. Notably, the FNIH criteria have only been validated in adults over 65 years, which is problematic when the mean life-expectancy of South Africans is 65.1 years [29]. To reduce discrepancies when reporting sarcopenia prevalence, African population-specific criterion needs to be used, and obesity adjusted for.

Sarcopenic obesity has been shown to exacerbate functional limitations and cardiometabolic risk [6, 19]. The current study showed that 38% of women reported that they had fallen in the past year, compared to 26.4% previously reported [52]. While a recent meta-analysis identified a higher rate of falls and fractures in older adults with sarcopenia [53], we show that those with sarcopenia had lower cardiovascular fitness and gait speed, but a similar prevalence of falls to those without sarcopenia. We suggest that collecting information on the number of falls may be more sensitive in detecting frailty and risk of sarcopenia. Further, environmental context is also important when understanding the mechanisms of falls in low-income settings (poor infrastructure, overcrowding and hazards in small dwellings) [52]. We also showed that 91% of women had multimorbidity (> 2 chronic diseases), with the three most prevalent diseases being hypertension (86%), dyslipidemia (73%) and type 2 diabetes (36%), which are all components of the metabolic syndrome. Although hypertension was the only disease that was more prevalent in those with sarcopenia, only waist circumference and triglycerides were the metabolic syndrome components that discriminated between those with and without sarcopenic obesity. Our results show that the high rate of chronic disease and multi-morbidity is of concern in this cohort and is probably reflective of the high prevalence of obesity. This demonstrates the need for targeting obesity and non-communicable diseases in LMIC settings.

Low-grade chronic inflammatory status is associated with obesity and sarcopenia and may represent increased risk for developing cardiometabolic diseases [20, 22, 54]. The current study showed that higher levels of systemic inflammation (as indexed by CRP) were associated with sarcopenic obesity, which is supported by a recent meta-analysis that showed that sarcopenia is associated with higher CRP, but not with higher IL-6 or TNFα [22]. Notably, CRP was associated with lower grip strength, but not muscle mass, which suggests that the inflammatory contribution to sarcopenic obesity may reflect muscle quality rather than quantity. Indeed, studies in older men and women (> 60 years) reported that higher baseline levels of IL-6 and CRP increased the risk for loss of strength over 3 years [55]. Accordingly, higher systemic concentrations of CRP may reflect higher adiposity in those with sarcopenia or sarcopenic obesity [19]. Women have higher relative adipose tissue compared to men and this may also explain previous data showing an independent association between low grade systemic inflammation (as indexed by CRP) and muscle strength in older women, but not men [56]. Consequently, sarcopenia may originate from processes such as age-related changes in body fat and body fat distribution, with the consequent low-grade chronic inflammatory state suggested to exacerbate progression of the disease [22].

Our results showed that a more food secure environment and financially supporting more children were part of the characteristic profile that described the sarcopenic obese cohort. National data from SA reports that 26% of the population regularly experience hunger and a further 28% are at risk of hunger, with access to food, household income and social protection (i.e. social grants for child support) all determining factors of food security [17]. Furthermore, socioeconomic status affects behavioural (i.e. PA and diet) characteristics [17, 18]. The present study shows an association between sarcopenic obesity and increased food security, suggesting a higher socioeconomic status, but lower PA and higher sedentary behaviour profile. Although dietary variables did not discriminate between those with and without sarcopenic obesity, we paradoxically showed that those who had a lower grip strength consumed more animal protein foods and less cooked porridge. Although these findings are difficult to explain, we hypothesise that either 1) the animal protein foods were of poor quality (i.e. processed meats, meat cuts with high fat content) and/or 2) those sufficiently affluent to buy meat and animal based products did less daily PA, which may impact on strength. However, longitudinal data is required to assess these hypotheses. Specifically, additional sources of high-quality protein that are sustainable, affordable and culturally appropriate for low-income communities (i.e., red meat, poultry, fish, dairy, soy, nuts seeds and legumes) in combination with a physically active lifestyle may preserve muscle mass and function in older adults [57, 58]. In a low-income setting, plant-based protein is the most affordable source and further research is required to determine the effects it has on the overall prevention of sarcopenia and sarcopenic obesity in these high-risk communities.

Socioeconomic status within low-income communities may influence PA patterns and overall cardiovascular fitness [17, 18, 59]. Previous research in older adults in highly developed countries have shown increased risk for all-cause mortality with higher sedentary time [60], and accumulating a higher percentage of sedentary time in bouts of > 60 min [61]. The current study showed that increased sitting time in bouts of 60 min rather than total daily sitting time were characteristics of the sarcopenic obese participants and were related to a lower grip strength_BMI_. The odds of being abdominally obese increases by 6.8% up to 48% for each 60 min sedentary bout increment [62]. Therefore, sedentary behaviour may also reflect higher levels of adiposity rather than sarcopenia per se. Regardless, higher adiposity in the current study was clearly associated with characteristics of sarcopenia. When considering the impact of sedentary behaviour on health-related outcomes, time spent being physically active, regardless of intensity also needs to be considered [60]. Importantly, a maximal risk reduction for all-cause mortality in older adults is observed at 7500 steps/day, 375 min/day of light PA, or 24 min/day of MVPA [60, 63]. In the present study, the mean daily step count of those without sarcopenia (7574 steps/day) was above the daily recommended step count for older adults, while those with sarcopenia were below, with a mean daily step count of 6037 steps/day. Although both groups were below the recommended light PA and MVPA recommendations, the sarcopenic group spent ~ 30 min/day less time in total MVPA and light PA compared to those without sarcopenia. Accordingly, our results suggest that increasing PA, while interrupting sedentary time can be collectively targeted for intervention-based research for the overall prevention of sarcopenic obesity.

### Limitations

The current study presents several limitations that need to be considered when interpreting the results. The cross-sectional nature of the study does not allow an assessment of causation. Thus, longitudinal and interventional studies are required in this population to extend the current descriptive findings. Although the HFIAS has been validated for use in South Africa, limitations with the access component of the questionnaire have been identified and expectations of possible financial or food aid influence results towards a more affirmative response [64]. The convenience sampling included few sarcopenic participants without obesity; however, the obesity prevalence reflects national data of older SA women, but these data may not be reflective of women in SA without obesity. Further, this study attempted to recruit an equal number of men and women, however there were major challenges with the recruitment, retention and compliance of the men, resulting in the capturing of incomplete data on only *n* = 25 men. This is common in epidemiological studies in SA and represent a significant limitation to the present study and current literature [65, 66]. Accordingly, these data can only be extrapolated to older black SA women. Larger cohort studies are required across Africa to understand the burden that sarcopenia and sarcopenic obesity may pose on the ageing population in LMIC.

## Conclusions

Collectively, we propose that the criterion presented by the FNIH is a feasible option for classifying sarcopenia in older, obese African women. The high prevalence of sarcopenia, obesity and multimorbidity demonstrate the need for sustainable interventions in these communities to reduce the burden on the health care system and to ensure that quality of life is maintained with ageing. Accordingly, targeting PA and dietary behaviours at a younger age should be a focus in LMIC to not only prevent obesity related multi-morbidities, but also sarcopenia.

## Data Availability

The datasets used and/or analysed during the current study are available from the corresponding author on reasonable request.
